# Laurinterol, the Main *Smart Secondary Metabolite* Among Lauranes and Cyclolauranes

**DOI:** 10.3390/md24060222

**Published:** 2026-06-22

**Authors:** Sara García-Davis, Ana R. Díaz-Marrero, José J. Fernández

**Affiliations:** 1 Instituto Universitario de Bio-Orgánica Antonio González (IUBO AG), Universidad de La Laguna (ULL), Avenida Astrofísico Francisco Sánchez 2, 38206 La Laguna, Spain; 2 Instituto de Productos Naturales y Agrobiología (IPNA), Consejo Superior de Investigaciones Científicas (CSIC), Avenida Astrofísico Francisco Sánchez 3, 38206 La Laguna, Spain; 3 Biotecnología Marina, IUBO-ULL, Unidad Asociada al IPNA-CSIC, 38206 La Laguna, Spain; 4 Departamento de Química Orgánica, Universidad de La Laguna (ULL), Avenida Astrofísico Francisco Sánchez 3, 38206 La Laguna, Spain

**Keywords:** *Smart Secondary Metabolites*, laurinterol, *Laurencia*, sesquiterpenes, lauranes, cyclolauranes, drug discovery

## Abstract

Laurinterol, a halogenated sesquiterpene produced by red algae of the genus *Laurencia*, is one of the most characteristic compounds within the laurane and cyclolaurane families. This review compiles and examines current knowledge on laurinterol, integrating evidence on its occurrence, biosynthesis, biological activities, and structural features. Within a functional and ecological framework, laurinterol is proposed as an archetypal *Smart Secondary Metabolite* (*SSM*), a concept that reflects the convergence of structural singularity, high abundance within its biosynthetic context, broad biological activity, multi-target interactions, and ecological or chemotaxonomic relevance. This perspective highlights its role in adaptive processes within producing organisms and associated trophic networks. Laurinterol exhibits a broad bioactivity profile, including antimicrobial, antimycobacterial, cytotoxic, antiparasitic, enzyme inhibitory, antifouling, and insecticidal or repellent effects. Structure–activity relationship (SAR) studies remain limited and are mainly developed in specific models, particularly against *Naegleria fowleri*. The current intellectual property landscape related to laurinterol, including patent applications, granted patents, and technological development trends, is also examined. Overall, this review positions laurinterol as a structurally distinctive and functionally relevant marine metabolite within chemical ecology and marine natural products research.

## 1. Introduction

Marine organisms are recognized as a rich source of structurally unique natural products. This chemical diversity arises from the unique biotic and abiotic conditions of their habitats, which have driven the evolution of specialized secondary metabolites as adaptive mechanisms for survival under high pressure, limited nutrient availability, high salinity, and intense ecological competition [1,2,3]. Over seven decades of systematic research, more than 44,000 marine natural products have been characterized, representing approximately 6% of the over 700,000 natural products described globally [4,5].

Among marine producers, macroalgae are recognized as an important source of structurally diverse secondary metabolites exhibiting a broad spectrum of biological activities, including antitumor, anti-inflammatory, antimicrobial, antidiabetic, antiviral, antihypertensive, fat-lowering, and neuroprotective activities [6]. Within the three taxonomic groups of macroalgae—green (Chlorophyta), red (Rhodophyta) and brown (Ochrophyta and Phaeophyceae)—red seaweeds have consistently been identified as the most prolific producers of bioactive metabolites [7].

In particular, red algae of the genus *Laurencia* have attracted sustained attention due to their outstanding chemical richness. Species of this genus have yielded a wide array of secondary metabolites displaying antimicrobial, antifungal, antiparasitic, cytotoxic, and anti-inflammatory activities. Most metabolites isolated from *Laurencia* species, as well as from mollusks grazing on them, belong to the sesquiterpene, diterpene, triterpene, and C_15_ acetogenin classes [8], highlighting both their biosynthetic specialization and ecological relevance.

Within this chemical repertoire, lauranes, cyclolauranes, and related metabolites constitute the second most frequently encountered class of sesquiterpenes reported from *Laurencia* spp. and *Aplysia* spp., with more than 100 compounds described to date. These metabolites are characterized by a 1,2,3-trisubstituted cyclopentane moiety and a 1,2-dimethylbicyclo[3.1.0]hexane system, both connected through a single bond to a six-membered ring, usually aromatic. Bromination at C-10 and/or C-12 and oxygenation at C-3 are common structural features. Among this group, laurinterol, isolaurinterol, debromolaurinterol, debromoisolaurinterol, laurene, isolaurene, allolaurinterol, debromoallolaurinterol, laurenisol, isolaurenisol, aplysin, debromoaplysin, aplysinol, and filiformins are the most frequently reported compounds from both *Laurencia* and *Aplysia* species [8].

**Figure 1:**
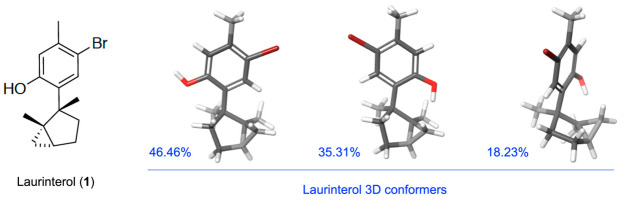
Structure of laurinterol and minimized 3D conformers.

Laurinterol (Figure 1), a brominated sesquiterpene first isolated in 1966 [9], has remained the subject of continuous investigation due to its abundance and functional versatility. It has been reported from multiple *Laurencia* species and from gastropods feeding on them, in some cases accounting for more than 20% of the crude extract. Laurinterol exhibits a wide range of biological activities, including antibacterial, antifungal, antiparasitic, cytotoxic, insecticidal, repellent, and antifouling effects, mediated through interactions with different molecular targets and mechanisms of action.

Taken together, these features support the consideration of laurinterol as a *Smart Secondary Metabolite* (*SSM*) within a functional and ecological framework. Here, the term *smart* metabolite is used to describe secondary metabolites that combine: (1) a singular chemical structure, (2) a high abundance among related metabolites, (3) potent biological activities, (4) the ability to interact with different biological targets, and (5) a significant ecological or chemotaxonomic value [10,11]. This concept reflects the convergence of structural, biosynthetic, functional, and ecological attributes that confer adaptive advantage to the producing organisms and their associated trophic networks.

## 2. Laurinterol as an Archetype of Smart Secondary Metabolites

The concept of a *Smart Secondary Metabolite* (SSM) has been proposed to designate certain natural metabolites that exhibit structural originality, biosynthetic adaptability, and a broad ability to interact with biological systems. Although this concept has been primarily illustrated through the study of elatol, a halogenated sesquiterpene characteristic of the genus *Laurencia*, these same features justify considering laurinterol as a SSM as well [10,11].

First, laurinterol stands out for its structural originality, as it is a halogenated sesquiterpene produced by species of the genus *Laurencia*. These algae accumulate halogenated metabolites in cortical inclusions known as *corps en cerise*, where compounds such as laurinterol and its derivative debromolaurinterol are found in high concentrations. The presence of halogens and the complexity of its terpenoid skeleton give laurinterol unique physicochemical properties within the *SSM* framework.

**Figure 2:**
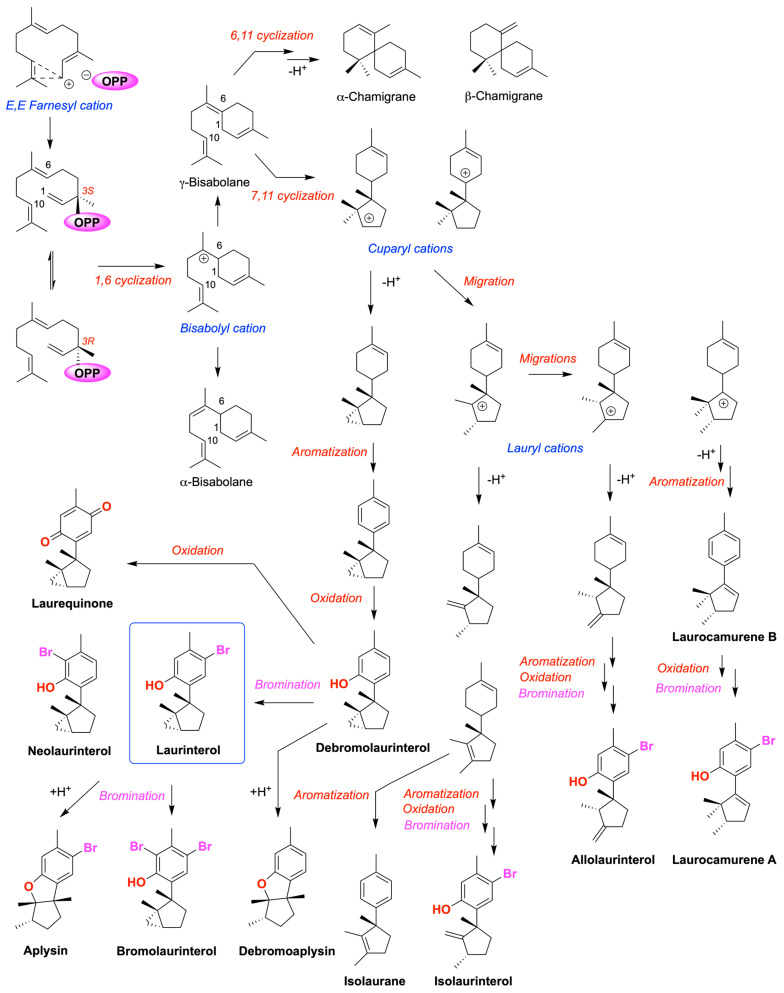
Biosynthetic pathway for laurinterol and related compounds.

Additionally, biosynthetic adaptability is a key criterion of the SSM concept. Although the biosynthetic pathway of laurinterol is not as well described as that of elatol, it is known that sesquiterpenes from *Laurencia* share complex cyclization and halogenation processes. Furthermore, the production of laurinterol (**1**) and debromolaurinterol (**2**) (Figure 2) can vary in response to environmental factors such as salinity changes, reflecting metabolic plasticity. This ability to generate chemical derivatives and modify their abundance depending on the environment aligns with the principle of biosynthetic flexibility proposed for SSM.

The third criterion is the broad capacity for biological interaction. Laurinterol exhibits well-documented bioactivities: it has antimicrobial and cytotoxic properties, already positioning it as a metabolite with relevant ecological functions in algal chemical defense. Even more significant is the recent discovery of its potent activity against *Naegleria fowleri*, a pathogenic protist responsible for primary amoebic meningoencephalitis. In vitro studies show that laurinterol not only exhibits potent amoebicidal activity against this amoeba at low concentrations but also induces programmed cell death and acts as a strong ATPase inhibitor, demonstrating far greater efficacy than other known inhibitors [12]. This broad range of interactions with essential cellular pathways aligns precisely with the definition of a *smart* metabolite capable of modulating diverse and impactful biological processes.

Finally, its ecological relevance must also be considered. Similar to other halogenated sesquiterpenes from *Laurencia*, laurinterol plays defensive roles within the alga’s ecology, contributing to protection against microorganisms and possibly influencing trophic or symbiotic interactions [13,14]. These complex ecological functions reinforce its classification within the category of metabolites that not only possess biological activity but also act as dynamic components in marine ecological networks.

Taken together, the structural originality, biosynthetic plasticity, and broad spectrum of biological activities support the conclusion that laurinterol clearly meets the criteria of a SSM, establishing itself as a versatile, adaptive, and highly interactive compound within the chemical repertoire of the genus *Laurencia*.

## 3. Search Strategy for Studies on Laurinterol

A systematic literature search was conducted in ScienceDirect, Scopus, PubMed, and MarinLit to identify studies on laurinterol. In ScienceDirect, an initial total of 71 records was retrieved, which was reduced to 12 after the application of filters based on title, abstract, and keywords. To reduce the risk of omitting relevant studies, an additional manual screening was performed, through which 28 articles were identified as eligible. These studies addressed identification, isolation, biological activity, biosynthesis, and/or synthesis of the compound, while reviews and book chapters were excluded.

In Scopus, 52 records were initially retrieved, of which 46 were retained after the application of the same filtering criteria. In PubMed, 15 records were identified through a general search. The search was supported by the specialized database MarinLit, which retrieved 44 records.

Overall, records from Scopus (*n* = 46), PubMed (*n* = 15) and MarinLit (*n* = 44) were selected for full-text assessment, and their eligibility was evaluated according to the predefined inclusion criteria. The 28 records identified from ScienceDirect following manual screening were considered preliminarily eligible and were included in the qualitative synthesis.

## 4. Laurinterol: Occurrence, Abundance and Trophic Distribution

### 4.1. Reported Sources of Laurinterol and Its Commonly Associated Compounds

Laurinterol is a brominated cyclolaurane-type sesquiterpene widely distributed in marine red algae of the genus *Laurencia*, where it has been reported in multiple species, including *L. intermedia*, *L. okamurae* (*L. okamurai*), *L. nidifica*, *L. pacifica*, *L. microcladia*, *L. tristicha*, *L. obtusa*, *L. decidua*, and *L. johnstonii*. It has been isolated from specimens collected across diverse geographic regions, including Japan, Korea, China, Greece, Turkey, the United States, and Mexico. In addition, laurinterol has been identified in herbivorous sea hares (*Aplysia* spp.), reflecting its transfer across trophic levels. The compound was first reported in 1966 from *L. intermedia*, together with debromolaurinterol, and was proposed as a biosynthetic precursor of aplysin [9].

Across *Laurencia* species, laurinterol consistently co-occurs with structurally related halogenated sesquiterpenes, particularly debromolaurinterol, isolaurinterol, and aplysin-type metabolites. This pattern is recurrent in species such as *L. intermedia*, *L. nidifica*, *L. pacifica*, and *L. johnstonii*, suggesting a conserved biosynthetic framework within the genus.

In species such as *L. okamurae* and *L. tristicha*, laurinterol is part of a broader metabolite assemblage that includes cuparanes, chamigrenes, C_15_-acetogenins, and other halogenated terpenoids. Despite this diversity, laurinterol remains a recurrent and often prominent component of the metabolite profile.

The occurrence of laurinterol in *Aplysia* species, together with related compounds such as aplysin, debromoaplysin, and pacifenol, further supports its ecological persistence and suggests a role in chemical defense mediated through dietary acquisition.

A detailed compilation of reported sources, associated metabolites, and quantitative data is provided in Table S1.

### 4.2. Content and Variability of Laurinterol in Laurencia Species

Although quantitative yields are not consistently reported, laurinterol is frequently described as a major metabolite when detected in species of the red algal genus *Laurencia*, often accounting for more than 20% of the crude extract [13,15,16,17,18,19]. In specimens of the sea hare *Aplysia*, however, laurinterol is not always the principal compound, although it commonly occurs among the dominant constituents. It should also be noted that laurinterol is often distributed across multiple chromatographic fractions in varying proportions. Moreover, because many studies prioritize structural identification over quantitative recovery, the compound is frequently detected but not exhaustively isolated, which may lead to an underestimation of its actual abundance.

From early studies, halogenated secondary metabolites in *Laurencia* were considered useful chemotaxonomic markers [20]. However, accumulated evidence indicates that laurinterol is not restricted to a single species but is instead broadly distributed across the genus. In most cases where it has been reported, it represents one of the major components of the extract, suggesting that its production reflects a shared metabolic feature rather than a strictly species-specific trait.

Quantitative analyses nevertheless reveal variability in laurinterol content across species and populations. For example, higher concentrations have been reported in *L. okamurai* (14.96–15.42 mg/g) compared to *L. tristicha* (5.31–9.15 mg/g), together with differences in the relative proportions of related metabolites such as debromolaurinterol and aplysinol [21]. Similarly, laurinterol may constitute a major fraction of the extract in some cases, while occurring at lower levels in others. These observations indicate that its relative abundance is variable, even when it is present as a characteristic metabolite.

Environmental factors appear to contribute to this variability. Experimental studies in cultured *L. okamurae* have shown that temperature and photoperiod have limited influence on laurinterol content, whereas increased salinity enhances its production. In contrast, growth under artificial culture conditions results in a marked decrease in metabolite levels [22], suggesting that natural environmental conditions play an important role in regulating its biosynthesis.

Overall, laurinterol should be regarded as a widely distributed and frequently dominant metabolite within *Laurencia*, whose abundance varies depending on both environmental conditions and biological context, rather than as a strictly species-specific compound.

## 5. Biosynthetic Pathway for Laurinterol and Related Compounds

The biosynthesis of laurinterol in red algae of the genus *Laurencia* begins with the formation of the isoprenoid precursors isopentenyl pyrophosphate (IPP) and dimethylallyl pyrophosphate (DMAPP) through the mevalonate (MVA) pathway. The presence of this pathway has been molecularly confirmed in species such as *Laurencia dendroidea*, where numerous genes associated with the MVA pathway have been identified [23]. IPP and DMAPP are subsequently condensed by prenyltransferases to yield farnesyl pyrophosphate (FPP), the central precursor for sesquiterpene biosynthesis. A sesquiterpene synthase (STS) then catalyzes the cyclization of FPP to generate a highly reactive carbocation intermediate. In *Laurencia*, STS-mediated cyclizations produce a broad range of complex sesquiterpene skeletons, which may further rearrange through Wagner–Meerwein migrations, a hallmark process in terpenoid biosynthesis (Figure 2). Transcriptomic analyses have identified at least 21 STS genes in *Laurencia*, supporting the extensive structural diversity observed within its terpenoid metabolome. Although the precise biosynthetic route to laurinterol has not been fully elucidated, current evidence suggests that it arises from a γ-bisabolane-type carbocation that undergoes sequential cyclizations and rearrangements to form the bicyclic cuparyl cation precursor.

Following the generation of the cuparane skeleton, additional enzymatic transformations give rise to the final structure of laurinterol. The intermediate undergoes dehydrogenation reactions catalyzed by oxidoreductases, which remove hydrogen atoms and enhance π-conjugation within the system. These stepwise transformations facilitate the aromatization of the terpene backbone, ultimately yielding the characteristic aromatic core of this metabolite. Subsequent halogenation steps, mediated by vanadium-dependent haloperoxidases, introduce halogen atoms at specific positions of the scaffold [24]. In parallel, other oxidoreductases participate in oxidation and functionalization processes, promoting hydroxylation and rearrangements that lead to the formation of laurinterol and structurally related sesquiterpenes, including neolaurinterol and laurequinone. Although the specific genes involved in these late-stage tailoring reactions remain uncharacterized, terpenome analyses in *Laurencia* reveal a complex and versatile enzymatic repertoire capable of supporting an extensive diversity of post-cyclization modifications [23].

Altogether, these recent discoveries underscore the remarkable metabolic plasticity of red algae. The ability to generate multiple halogenated derivatives from a single terpenoid backbone—now supported by the functional characterization of additional type I TSs—highlights the chemical innovation inherent to *Laurencia* and related genera [25].

## 6. Biological Activities and Action Modes

**Figure 3:**
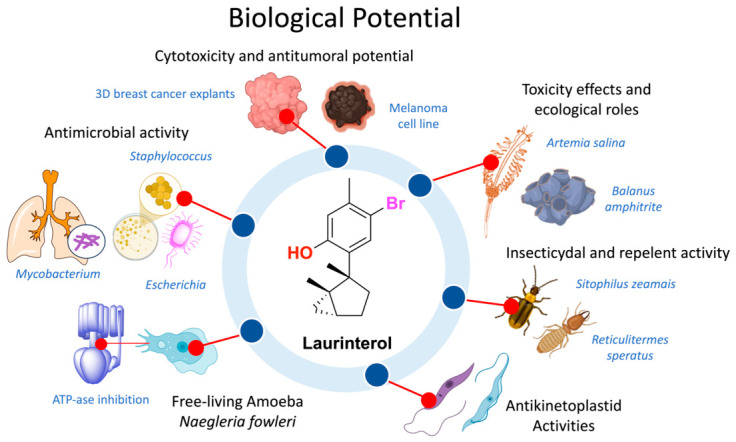
Scheme of the biological effects of laurinterol.

Laurinterol exhibits a broad spectrum of biological activities, as summarized in Figure 3 and Table S2. It demonstrates potent antimicrobial effects against pathogenic bacteria (including antibiotic-resistant strains) and marine biofilm-forming *Bacillus* species. Antimycobacterial activity is also prominent against *M. tuberculosis* strains and nontuberculous mycobacteria. Cytotoxic potential spans multiple cancer cell lines, alongside antiparasitic effects against *Naegleria fowleri* and *Leishmania amazonensis*. Additional activities include Na,K-ATPase inhibition, acetylcholinesterase inhibition, antifouling properties and insecticidal/repellent effects.

### 6.1. Toxicity Effects

Based on the available data, laurinterol exhibits a differential toxicity profile depending on the biological model evaluated. In the *Artemia salina* bioassay, it has shown moderate acute toxicity, with an LC_50_ value of 4.14 μg/mL [26], indicating significant biological activity against this organism, commonly used as a general bioindicator of baseline toxicity rather than a specific biological target. In contrast, no relevant toxic effects were observed on nauplii of the barnacle *Balanus amphitrite*, as the EC_50_ value was greater than 100 μg/mL [27], suggesting low susceptibility in this marine model. In murine macrophages, laurinterol displayed moderate cytotoxicity, with a CC_50_ of 80.11 μM [19], indicating a relatively wide cellular safety margin at low to moderate concentrations. Overall, these results suggest that laurinterol presents selective toxicity, which is relevant for its further evaluation in biotechnological and pharmacological applications.

Collectively, these findings indicate that laurinterol exhibits a context-dependent toxicity profile, characterized by measurable activity in generalized bioindicator assays such as *Artemia salina*, limited effects on certain marine invertebrate models, and moderate cytotoxicity in mammalian cells. The variability in susceptibility among biological systems suggests that its toxic effects are not indiscriminate but instead influenced by organism-specific physiological features, exposure conditions, and possibly differential membrane composition or metabolic capacity. Nevertheless, the current evidence remains primarily based on in vitro or simplified bioassays, underscoring the need for more comprehensive toxicological evaluations to fully define its safety profile and therapeutic window.

### 6.2. Antimicrobial Activity

Laurinterol has demonstrated pronounced activity against several Gram-positive bacteria. Strong inhibitory effects were initially reported against *Staphylococcus aureus*, with effective concentrations ranging from 1 to 5 μg/mL [28]. Consistent with these findings, disk diffusion assays showed an inhibition zone of 12 mm against *S. aureus* at 25 μg/disc [29].

In addition, laurinterol exhibited potent activity against marine-associated bacteria, biofilm-forming Gram-positive bacteria of the genus *Bacillus*, including *B. altitudinis*, *B. pumilus*, *B. subtilis*, and *B. cereus*, with MIC values below 3.9 μg/mL [30]. These results suggest a potential ecological role in inhibiting bacterial colonization and biofilm formation on algal surfaces.

Laurinterol also displays inhibitory activity against several Gram-negative species, although potency varies among strains. Reported targets include *Alteromonas* sp., *Azomonas agilis*, *Erwinia amylovora*, and *Escherichia coli*, with MIC values ranging from 5 to 10 μg/disc, while slightly lower activity was observed against *Azotobacter beijerinckii* (MIC = 15 μg/disc) [13].

Further evaluation against a broader panel of 22 pathogenic bacterial strains, including seven antibiotic-resistant isolates, revealed low MIC values between 1.56 and 6.25 μg/mL [31], underscoring the compound’s activity across taxonomically diverse bacteria and its potential relevance in antimicrobial resistance contexts.

Laurinterol has also been evaluated against mycobacterial species. Early studies reported strong activity against *Mycobacterium smegmatis*, with effective concentrations in the range of 1–5 μg/mL [28]. Subsequent investigations demonstrated activity against clinically relevant species. In *Mycobacterium tuberculosis*, MIC values ranged from 25 to 100 μg/mL across eight strains, whereas higher potency was observed against six nontuberculous mycobacterial species, with MIC values between 6.2 and 25 μg/mL [32]. These findings indicate selective antimycobacterial activity within the genus, with variable susceptibility among species.

Moderate antifungal activity has been reported against the yeast *Candida albicans*, with effective concentrations between 10 and 100 μg/mL [28], indicating that laurinterol’s antimicrobial spectrum extends to eukaryotic microorganisms.

Taken together, the antimicrobial profile of laurinterol reveals activity across taxonomically diverse microorganisms, including Gram-positive and Gram-negative bacteria, mycobacteria, and fungi. The compound displays particularly notable potency against Gram-positive and selected marine-associated strains, as well as variable but consistent activity against clinically relevant pathogens.

### 6.3. Antiparasitic Activity

Laurinterol has demonstrated relevant antiparasitic activity against protozoan pathogens of medical importance, with evidence of both stage-dependent and multi-target effects. In *Leishmania amazonensis*, the compound exhibited comparable inhibitory activity against promastigote and intracellular amastigote stages, with IC_50_ values of 34.45 μM and 34.72 μM, respectively, indicating a consistent effect across the parasite life cycle [19].

**Figure 4:**
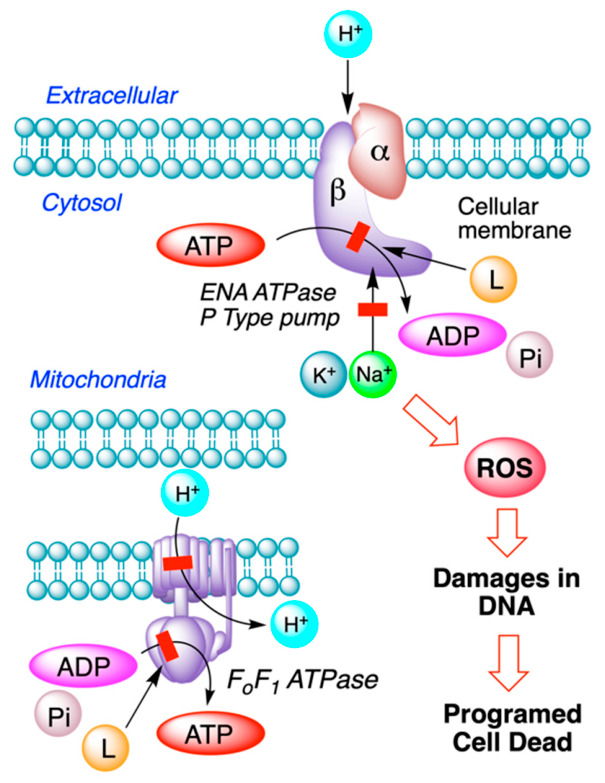
Laurinterol as an ATP competitive inhibitor of P-type ATPases in *Naegleria fowleri*. L represents laurinterol.

Notably, laurinterol showed higher potency against the free-living amoeba *Naegleria fowleri*. In vitro assays against the trophozoite stage revealed an IC_50_ value of 13.42 μM. Morphological and biochemical analyses indicated that laurinterol induces DNA condensation, membrane and mitochondrial damage, and reactive oxygen species (ROS) generation, all consistent with programmed cell death–like processes. Additionally, a marked depletion of intracellular ATP levels (97.36%) was observed, suggesting interference with energy metabolism. In this context, laurinterol has been proposed to act as an inhibitor of ATPase pumps, including the Na^+^/K^+^-ATPase, showing inhibitory effects at concentrations significantly lower than reference inhibitors such as furosemide. The lack of activity of the oxidized laurinterol dimer (IC_50_ > 100 μM) further supports the importance of its native structure for biological activity and suggests a competitive, but not highly specific, mode of enzyme inhibition (Figure 4) [12].

Laurinterol also exhibited significant activity against the cyst stage of *N. fowleri*, with an IC_50_ value of 8.81 μM [33]. Although cyst formation has not been definitively established in primary amoebic meningoencephalitis (PAM) patients, the ability to target this resistant stage is relevant, as encystation could contribute to treatment failure or recurrence.

Beyond its cytotoxic effects, laurinterol has been shown to interfere with key pathogenic processes. In adhesion assays, the compound significantly reduced trophozoite attachment to extracellular matrix (ECM) components, including laminin and Matrigel^®^, with rapid and near-complete inhibition observed at higher concentrations (85 μM). This fast-acting anti-adhesion effect suggests disruption of early host–pathogen interactions, potentially linked to membrane-associated mechanisms [34].

In contrast, laurinterol did not exhibit detectable activity against *Acanthamoeba castellanii* Neff at the concentration evaluated (100 μg/mL) [35], indicating that its antiparasitic effects are not universal across free-living amoebae and may depend on species-specific physiological or molecular features.

Supporting its pharmacological potential, in silico ADME/Tox analyses and molecular modeling studies have identified laurinterol and related cyclolaurane sesquiterpenes (e.g., debromolaurinterol and allolaurinterol) as promising scaffolds for the development of treatments against primary amoebic meningoencephalitis [33].

Taken together, these findings indicate that laurinterol displays consistent antiparasitic activity across distinct protozoan models, with comparable potency across life-cycle stages of *Leishmania amazonensis* and enhanced activity against both trophozoite and cyst forms of *Naegleria fowleri*. Its ability to affect multiple biological processes—including energy metabolism, membrane integrity, oxidative balance, and cell adhesion—suggests interaction with conserved cellular targets rather than stage-specific mechanisms. However, the lack of activity against *Acanthamoeba castellanii* highlights a degree of species-dependent selectivity. Although the observed IC_50_ values fall within the low-to-moderate micromolar range, the functional breadth of its activity underscores the need for further mechanistic and selectivity studies.

### 6.4. Cytotoxic and Antitumoral Activity

Laurinterol has demonstrated cytotoxic activity against a diverse panel of human tumor cell lines, although potency varies considerably depending on the cellular model and experimental conditions. In an initial screening, EC_50_ values ranging from 2.4 to 12.6 μg/mL were reported against A549 (non-small cell lung adenocarcinoma), SK-OV-3 (ovarian carcinoma), SK-MEL-2 (melanoma), XF498 (central nervous system cancer), and HT15 (colon carcinoma) cell lines, indicating relatively strong growth-inhibitory effects in these models [36]. Moderate activity was also observed against HeLa (cervical adenocarcinoma) cells, with an IC_50_ of 32 μg/mL [29].

In contrast, other evaluations have reported IC_50_ values in the micromolar range (67.2–165.8 μM) against a broader panel, including CHO (non-tumoral ovarian cells), K562 (chronic myelogenous leukemia), MCF7 (breast adenocarcinoma), PC3 (prostate adenocarcinoma), HeLa, A431 (epidermoid carcinoma), A549, and NSCLC-N6 (lung cancer) cell lines, suggesting moderate cytotoxic potency under those experimental settings [37]. Additional data indicate IC_50_ values of 15.68 μg/mL for Vero cells and 16.07 μg/mL for MCF-7 cells [38], supporting a biologically relevant but not uniformly high cytotoxic effect.

**Figure 5:**
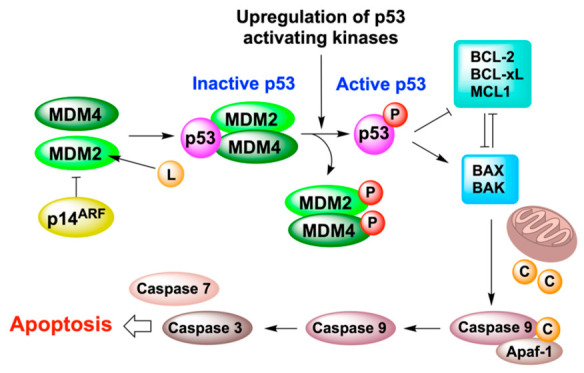
Proposed mechanism of apoptosis for laurinterol (L).

Mechanistic insights, although still limited, suggest that laurinterol-containing extracts may induce apoptosis through regulated pathways. In melanoma B16F1 cells, an extract of *Laurencia okamurai* containing laurinterol promoted apoptosis, as evidenced by DNA fragmentation, caspase activation, and cell cycle alterations. This effect was associated with transcriptional activation of p53 and p21, as well as increased levels of phosphorylated p53, indicating the involvement of a p53-dependent apoptotic pathway (Figure 5) [39].

Importantly, ex vivo assays using human breast cancer explants treated with laurinterol at 30 μg/mL revealed a heterogeneous response pattern. Sensitive tumor samples exhibited marked necrotic cell death, whereas resistant samples showed no detectable toxic effects [38]. This variability suggests that laurinterol’s antitumoral activity may depend on tumor-specific molecular or metabolic characteristics rather than exerting indiscriminate cytotoxicity.

Overall, the available evidence indicates that laurinterol possesses moderate and cell line–dependent cytotoxic activity, with stronger effects in certain epithelial-derived cancers. The induction of apoptosis through pathways involving p53 highlights a potential mechanism of action, although this has been demonstrated in extract-based systems and requires confirmation with the pure compound. Variability across experimental models and limited mechanistic resolution underscore the need for further studies to define its molecular targets, mode of action, and therapeutic selectivity relative to non-tumoral cells.

### 6.5. Miscellaneous Biological Activities

Beyond its antimicrobial, antiparasitic, and cytotoxic properties, laurinterol exhibits additional biological activities that reflect its broad ecological and biochemical relevance.

Antiviral activity. In Huh7 GL4.18 CURS_BC_AeUS reporter cells, which express firefly luciferase under the control of the hepatitis B virus (HBV) core promoter (CURS–BCP), laurinterol exhibited inhibitory activity with an EC_50_ value of 25.5 μM [40]. This result suggests a potential modulatory effect on viral transcriptional regulation, although the underlying mechanism remains to be elucidated.

Insecticidal activity. Laurinterol showed potent insecticidal effects against the termite *Reticulitermes speratus*, with an LD_50_ value of 2.20 μg/insect, indicating strong acute toxicity in this model [26]. This level of activity suggests potential interference with essential physiological pathways in insects and supports its possible ecological role as a chemical defense metabolite.

Larvicidal and oviposition deterrent activity. The ethanolic extract of *Laurencia johnstonii* exhibited larvicidal activity against third-instar larvae of *Aedes aegypti*, with the most active fraction showing an LC_50_ of 240 μg/mL. This fraction contained a mixture of sesquiterpenes, with laurinterol identified as the major component, suggesting its contribution to the observed activity. However, the effect was not evaluated for the pure compound. The fraction also showed acetylcholinesterase inhibitory activity, indicating a possible mechanism of action [41].

Repellent activity. In behavioral assays against the maize weevil *Sitophilus zeamais*, laurinterol displayed moderate repellent activity, with an ED_50_ of 12.65 μg/cm^2^ [26]. Although less potent than its insecticidal effect, this activity reinforces its potential function in deterrence against herbivorous or pest species.

Antibiofilm activity. Laurinterol has been reported as a potent antibiofilm agent, capable of reducing more than 97% of biofilm formation in marine biofilm-forming bacteria, including *Bacillus altitudinis*, *B. pumilus*, *B. subtilis*, and *B. cereus* [30].

Antifouling activity. Laurinterol has demonstrated remarkable potency in preventing larval settlement of the barnacle *Balanus amphitrite*, with an EC_50_ value of 0.27 μg/mL. This low effective concentration highlights its strong antifouling capacity and supports its potential application in environmentally friendly antifouling strategies, particularly in the development of non-toxic marine coatings [27]. Consistent results have been reported in related models, where laurinterol exhibited activity against larvae of *Amphibalanus amphitrite,* with an EC_50_ of 0.65 μg/mL and an LC_50_ of 5.8 μg/mL [42]. Additional studies have reported effects in the micromolar range, including IC_50_ (settlement), LC_50_ (mortality) and EC_50_ (inhibition of metamorphosis) values of 2.22, 5.29 and 10,85 μM at 48 h, respectively [43]. More recently, environmentally friendly fishing nets incorporating laurinterol into hydrogel-based polymer systems, such as polyvinyl alcohol (PVA) and poly(methacrylic acid) [poly(MAAc)], have demonstrated remarkable antifouling performance, suppressing byssal thread formation by up to 93% [44].

Enzymatic inhibition. At the biochemical level, laurinterol has been reported to inhibit acetylcholinesterase, with IC_50_ values of 46.85 and 59 μg/mL in independent assays, indicating moderate inhibitory activity [26,41]. Additionally, it inhibits Na^+^/K^+^-ATPase with an IC_50_ of 0.04 mM, suggesting potential effects on ion transport and membrane-associated processes [45]. These enzymatic activities may partially underlie some of its observed bioactivities, including insecticidal or cytotoxic effects.

Collectively, these miscellaneous activities further illustrate the functional versatility of laurinterol across ecological, biochemical, and applied contexts. The diversity of targets affected—ranging from invertebrate physiology to membrane-associated enzymes—supports the view of laurinterol as a multifunctional secondary metabolite, while emphasizing the need for mechanistic studies to clarify whether these activities arise from specific molecular interactions or broader membrane-related effects.

## 7. Structure–Activity Relationships (SAR)

Comparative studies between laurinterol and related cyclolaurane sesquiterpenes provide a clear framework to define SAR, particularly against *Naegleria fowleri*. Laurinterol and debromolaurinterol differ only in the presence of a bromine atom, constituting a minimal and informative model for assessing the role of halogenation.

Both compounds share a common mechanism characterized by a strong depletion of intracellular ATP levels and collapse of the mitochondrial membrane potential, consistent with ATPase inhibition and disruption of energy metabolism. Debromolaurinterol induces a slightly greater ATP reduction (99.98%) than laurinterol (97.36%), indicating that dehalogenation does not abolish activity at the cellular level. However, enzymatic assays show that laurinterol is a more potent inhibitor of Na^+^/K^+^-ATPase (IC_50_ = 40 μM) than debromolaurinterol (IC_50_ = 400 μM), suggesting that bromination enhances target-specific interactions [12]. These differences are also reflected in antiparasitic potency and selectivity. Laurinterol exhibits the highest activity against *N. fowleri* (IC_50_ = 13.42 μM) combined with relatively low cytotoxicity (CC_50_ = 80.11 μM), resulting in a favorable selectivity profile. In contrast, debromolaurinterol shows reduced potency (IC_50_ = 18.76 μM) and slightly higher cytotoxicity (CC_50_ = 70.13 μM), while isolaurinterol displays both lower antiparasitic activity (IC_50_ = 28.18 μM) and significantly higher cytotoxicity (CC_50_ = 24.74 μM). These trends indicate that both halogenation and subtle structural variations within the cyclolaurane scaffold influence not only potency but also selectivity toward parasitic versus mammalian cells [12]. Computational analyses further support these observations. Differences in lipophilicity (log P: 3.81 for debromolaurinterol, 4.32 for laurinterol, and 4.46 for allolaurinterol) indicate that bromination increases hydrophobicity, which may enhance membrane permeability and molecular interactions. All compounds exhibit favorable predicted ADME/Tox profiles, supporting the cyclolaurane scaffold as a viable pharmacophore [12].

Overall, the activity of laurinterol and related metabolites is governed by a balance between halogenation, which enhances target interaction and selectivity, and structural features that preserve molecular recognition. These findings support a mechanism involving ATP-competitive inhibition and reinforce the cyclolaurane scaffold as a promising basis for antiparasitic drug development.

The SAR trends observed against *N. fowleri* are also supported by studies conducted in other biological models. Comparative evaluations of laurinterol, isolaurinterol, debromolaurinterol, and aplysin reveal that relatively small structural modifications can substantially alter biological activity. For example, laurinterol consistently displays broader antibacterial activity than isolaurinterol against marine bacterial strains [13], suggesting that stereochemical differences affect target recognition and antimicrobial potency. Similar effects are observed in antiparasitic assays, where laurinterol exhibits greater activity and lower cytotoxicity than isolaurinterol against *N. fowleri*. In contrast, debromolaurinterol retains comparable antiamoebic activity, indicating that bromination is not essential for activity per se, although it contributes to target-specific interactions and selectivity [12].

Additional evidence comes from antileishmanial studies, where debromolaurinterol and isolaurinterol display enhanced activity against *Leishmania amazonensis* promastigotes relative to laurinterol, whereas isolaurinterol simultaneously exhibits increased cytotoxicity toward mammalian cells [19]. These observations suggest that both bromination and stereochemistry influence the balance between potency and selectivity. Comparisons between laurinterol and aplysin further demonstrate the importance of the overall molecular framework. Despite their close biosynthetic relationship, the structural rearrangement from the cyclolaurane skeleton of laurinterol to the laurane skeleton of aplysin is associated with a marked reduction in antibacterial, antimycobacterial, and antiparasitic activities. This trend is particularly evident against *Mycobacterium tuberculosis* and non-tuberculous mycobacteria, where laurinterol consistently exhibits greater potency and a broader activity spectrum [32]. Interestingly, the reduced biological activity of aplysin is often accompanied by lower cytotoxicity, indicating that skeletal rearrangement influences both efficacy and safety profiles.

Taken together, these studies indicate that biological activity within the laurane/cyclolaurane family depends on the interplay of multiple structural features rather than on a single pharmacophoric element. Bromination, stereochemical arrangement, and the overall molecular architecture all contribute to modulating potency, selectivity, and target engagement across different biological systems.

## 8. Intellectual Property

Research on laurinterol has generated increasing biotechnological and pharmacological interest, as reflected in several patent applications focused on its isolation, characterization, and therapeutic potential. These inventions primarily explore its use as a bioactive compound, especially within the field of oncology.

One of the most prominent patents is WO2009048195A1 / KR20090036236A [46,47], entitled “Laurinterol compound derived from *Laurencia okamurai* for the prevention and inhibition of melanoma”. This international application and its corresponding South Korean national patent describe the use of laurinterol isolated from *L. okamurae* as the active ingredient in pharmaceutical compositions designed to prevent or inhibit melanoma cell growth, based on its ability to induce apoptosis in tumor cells and thereby inhibit cancer cell proliferation. Accordingly, the patents include claims concerning formulations containing laurinterol at concentrations ranging from 0.1 to 1000 µg/mL for melanoma treatment or prevention, while also highlighting its potential advantages over certain synthetic drugs due to reduced adverse effects.

Beyond patents specifically addressing anticancer applications, other inventions broaden the scope of laurinterol and structurally related compounds. The patent EP2198713A1 [48], although not exclusively centered on laurinterol, describes organic extracts obtained from *Laurencia* species containing multiple bioactive metabolites, including aromatic halogenated sesquiterpenes structurally related to laurinterol. This invention is oriented toward antifouling applications, demonstrating the functional versatility of compounds present in these algae and their potential in marine and environmental biotechnology.

In addition, pharmaceutical databases include references to structural derivatives of laurinterol, such as allolaurinterol, which has been investigated for its inhibitory activity on translation initiation factors, particularly eIF4A. Although this derivative is not the subject of a dedicated patent, its presence in specialized repositories underscores the growing recognition of halogenated sesquiterpenes from *Laurencia* as promising chemical scaffolds for future therapeutic development, extending their relevance beyond oncology.

Overall, the landscape of patents related to laurinterol reflects a sustained and expanding interest in both its pharmacological applications, especially as an antitumor agent, and its broader biotechnological potential. The diversity of approaches, ranging from formulations based on pure compounds to complex extracts with multiple biological activities, highlights the significance of laurinterol as one of the most valuable natural products derived from the genus *Laurencia* and a strategic resource for the development of novel therapeutic and bioindustrial technologies.

## 9. Conclusions

Laurinterol emerges as a robust example of a *Smart Secondary Metabolite* (*SSM*), supported by convergent evidence of structural singularity, high abundance within its biosynthetic context, broad and consistent bioactivity, and documented ecological and chemotaxonomic relevance. Rather than representing isolated properties, these features collectively reflect a metabolite with functional coherence across biochemical, ecological, and evolutionary dimensions.

Among its reported biological properties, antiparasitic, antibacterial, and cytotoxic activities stand out as particularly promising, highlighting its potential as a lead compound for the development of new therapeutic agents. Importantly, the structural simplicity and synthetic accessibility of laurinterol, together with the existence of numerous natural and synthetic derivatives, make it an attractive chemical scaffold for the rational design of novel bioactive molecules.

Although significant progress has been made in characterizing its biological effects, the molecular mechanisms underlying many of these activities remain only partially understood. The ability of laurinterol to interact with different biological targets suggests a complex pharmacological profile that deserves deeper investigation through mechanistic studies, target identification approaches, and structure–activity relationship analyses. Such efforts may reveal new opportunities for optimizing its biological properties and expanding its therapeutic potential.

This review contributes to the field by integrating, for the first time, the available evidence on the biological activities, mechanisms of action, ecological significance, chemotaxonomic value, and intellectual property landscape of laurinterol within the SSM framework. By identifying both established knowledge and critical research gaps, this work provides a foundation for future studies aimed at understanding the functional role of laurinterol and exploiting its potential as a model compound for marine natural product research and drug discovery.

## Data Availability

No new data were created or analyzed in this study. Data sharing is not applicable to this article.

## Supplementary Materials

The following supporting information can be downloaded at: https://www.mdpi.com/article/10.3390/md24060222/s1, Table S1: Sources of laurinterol and related compounds [9,12,13,15,16,17,18,19,27,29,35,37,38,49,50,51,52,53,54,55,56,57,58,59,60,61,62,63,64,65,66,67]; Table S2: Biological activities referenced for laurinterol [12,13,19,26,27,28,29,30,31,32,33,36,37,38,40,41,42,44,45].
