# Supplementary material for: Laurinterol, the Main Smart Secondary Metabolite Among Lauranes and Cyclolauranes
Source: Mar Drugs. 2026 Jun 22;24(6):222. doi: 10.3390/md24060222 (PMC13301172; doi:10.3390/md24060222)
Supplement: Supplementary file 1 [file marinedrugs-24-00222-s001.zip › marinedrugs-4350118-supplementary.pdf]

Supplementary Materials

Laurinterol, The Main *Smart Secondary Metabolite* among Lauranes and Cycloraluranes

Sara García-Davis[1], Ana R. Díaz-Marrero[2,3] and José J. Fernández [1,3,4]

[1] Instituto Universitario de Bio-Orgánica Antonio González (IUBO AG), Universidad de La Laguna (ULL), Avenida Astrofísico Francisco Sánchez 2, 38206 La Laguna, Spain; jjfercas@ull.edu.es
[2] Instituto de Productos Naturales y Agrobiología (IPNA), Consejo Superior de Investigaciones Científicas (CSIC), Avenida Astrofísico Francisco Sánchez 3, 38206 La Laguna, Spain; adiazmar@ipna.csic.es
[3] Biotecnología Marina, IUBO-ULL, Unidad Asociada al IPNA-CSIC, 38206 La Laguna, Spain
[4] Departamento de Química Orgánica, Universidad de La Laguna (ULL), Avenida Astrofísico Francisco Sánchez 3, 38206 La Laguna, Spain

Table S1. Sources of laurinterol and related compounds.

Source of laurinterol	Main co-occurrent compounds	Country of collection	References
L. intermedia	Debromolaurinterol<br>Isolaurinterol	Japan	**[9]** Irie *et al*. **1966** https://doi.org/10.1016/S0040-4039(00)90267-3<br>**[49]** Irie *et al*. **1970** https://doi.org/10.1016/S0040-4020(01)92906-0
L. okamurae (*L. okamurai*)	Debromolaurinterol, isolaurinterol, laurinterol acetate, debromoisolaurinterol, neolaurinterol, aplysin, debromoaplysin, isoaplysin, aplysinal, aplysinol, debromoaplysinol, 3α-hydrxydebromoaplysin, 3β-hydroxyaplysin, laurokamurene A, laurokamurene B, laurokomurenene A, dibromophenol, isoaplysin, bromocuparene, okamurallene, isobromocuparene, cuparene-type ether, isolaurene, α-bromocuparene, α-bromoisocuparene, pacifenol, prepacifenol, prepacifenol epoxide, bromoallene, johnstonol, neolaurencenyne, isolaurene, (-)-α-bromocuparene, laurequinone, nidificene, 1-deoxyprepacifenol, 4,10-dibromo-3-chloro-9-hydroxy-α-chamigrene, 4,10-dibromo-3-chloro-α-chamigrene, nanji A, 3α-hydroperoxy-3-epiaplysin, 3β-hydroperoxyaplysin, laurencenyne 5, neolaurencenyne 6	Japan, USA, Korea	**[50]** Irie *et al*. **1969** https://doi.org/10.1246/bcsj.42.843<br>**[16]** Suzuki and Kurosawa, **1978** https://doi.org/10.1016/S0040-4039(01)94811-7<br>**[17]** Suzuki and Kurosawa, **1979** https://doi.org/10.1246/bcsj.52.3352<br>**[51]** Suzuki and Kurosawa **1981** https://doi.org/10.1016/S0040-4039(01)91327-94039(01)91327-9<br>**[52]** Kigoshi *et al.* **1981** https://doi.org/10.1016/S0040-4039(01)83025-2<br>**[53]** Ojika *et al.* **1982** https://doi.org/10.1016/0031-9422(82)85220-5<br>**[18]** Suzuki and Kurosawa **1985** https://doi.org/10.1016/S0031-9422(00)83109-X<br>**[27]** Ryu and Yoon, **2003**<br>**[67]** Mao and Guo, **2006** https://doi.org/10.1021/np0503810<br>**[54]** Yang *et al.* **2018** https://doi.org/10.1016/j.bse.2018.05.003<br>**[55]** Minamida *et al.* **2021** https://doi.org/10.1016/j.bse.2021.104259
L. nidifica	Debromolaurinterol, isolaurinterol, aplysin, debromoaplysin, pacifenol, nidificene, nidifidiene, nidifidienol	USA, Japan	**[14]** Waraszkiewicz and Erickson **1974** https://doi.org/10.1016/S0040-4039(01)82616-2<br>**[56]** Waraszkiewicz and Erickson **1975** https://doi.org/10.1016/S0040-4039(00)71843-0<br>**[57]** Shizuri *et al.* **1984** https://doi.org/10.1016/S0031-9422(00)84124-2

			**[13]** Vairappan *et al*. **2001** https://doi.org/10.1016/S0031-9422(01)00260-6
L. pacifica	Debromolaurinterol, isolaurinterol, debromoisolaurinterol, pacifenol, pacifidiene, tridehalopacifenol	USA	**[58]** Sims *et al*. **1971** https://doi.org/10.1021/ja00744a041 **[59]** Howard *et al*. **1980** https://doi.org/10.1016/0305-1978(80)90035-6
L. microcladia	Bromolaurenisol	Grece	**[37]** Kladi *et al*. **2006** https://doi.org/10.1016/j.tet.2005.09.113
L. tristicha	Debromolaurinterol, isolaurinterol, isolaurene, isobromocuparene, debromoaplysin, aplysinol, debromoaplysinol, dimer of laurinterol, dibromophenol, laur-11en-1,10β-diol, 4-bromolaur-11-en-1,10α-diol, 4-bromolaur-11-en-1,10β-diol	China	**[60]** Ji *et al*. **2008** http://dx.doi.org/10.1080/14786410802019309
L. obtusa	Obtusenol, obtusenyne, Epibrasilenol, *cis*-isodihydrorhodophytin	Turkey	**[61]** Imre *et al*. **1981** https://doi.org/10.1016/0031-9422(81)85190-4
L. decidua	Isolaurinterol, aplysin, aplysinol	Mexico	**[62]** McMillan *et al*. **1976** https://doi.org/10.1016/0040-4039(76)80078-0
L. johnstonii	Isolaurinterol, aplysin, α-bromocuparane, α-isobromocuparane, debromolaurinterol, laurequinone	Mexico	**[35]** García-Davis *et al*. **2018** https://doi.org/10.3390/md16110443 **[38]** García-Davis *et al*. **2019** https://doi.org/10.3390/md17040201 **[12]** Arberas-Jiménez *et al*. **2020** https://doi.org/10.1038/s41598-020-74729-y **[19]** García-Davis *et al*. **2023** https://doi.org/10.3390/md21060333
Laurencia sp.	2,10-dibromo-3-chloro-α-chamigrene, bromoallene	Japan	**[63]** Suzuki *et al*. **2005** https://doi.org/10.1016/j.phytochem.2005.08.008
A. californica	Debromolaurinterol, aplysin, debromoaplysin, pacifenol, johnstonol pacifidiene, 3,7-dimethyl-l,8,8-tribromo-3,4,7-trichloro-l,5-octadiene, 7-chloro-3,7-dimethyl-l,4,6-tribromo-l-octen-3-ol,	USA	**[64]** Stallard and Faulkner, **1974** https://doi.org/10.1016/0305-0491(74)90218-1
A. kurodai	Laurinterol acetate, debromolaurinterol, debromolaurinterol acetate	Japan	**[29]** Tsukamoto *et al*. **2005** https://doi.org/10.3390/md302022
A. parvula	Debromolaurinterol, aplysin, aplyparvunin	Japan	**[65]** Miyamoto *et al*. **1995** https://doi.org/10.1016/0040-4039(95)01205-V
A. punctata	Laurene, α-bromocuparane, α-isobromocuparane, debromoallolaurinterol,	Italy	**[66]** Findlay and Li, **2002** https://doi.org/10.1139/v02-189

	laurenisol, isofiliformin, obtusenol β-snyderol, isoconncindiol, parguerol, laurenyne laurencenyneneoparguerol dione, deacetylparguerol, punctatene acetate, punctatol, perforenone, perforatol		

Table S2. Biological activities referenced for laurinterol.

Biological activity	Details	Reference
Toxicity	LC_{50} 4.14 µg/mL against *Artemia salina*	**[26]** Ishii *et al*. **2017**
	EC_{50} > 100 µg/mL against nauplii of the barnacle *B. amphitrite*	**[27]** Ryu and Yooh **2003**
	CC_{50} 80.11 µM against murine macrophages	**[19]** García-Davis *et al*. **2023** https://doi.org/10.3390/md21060333
Antimicrobial	Strong activity (1-5 µg/mL) against *S. aureus* and *M. smegmatis*; moderate (10-100 µg/mL) against *C. albicans*	**[28]** Sims *et al*. **1975** https://doi.org/10.1128/aac.7.3.320
	MIC 5-10 µg/disc against *Alteromonas* sp., *Azomonas agilis*, *Erwinia amylovora*, and *E. coli*; MIC 15 µg/disc against *Azotobacter beijerinckii*	**[13]** Vairappan *et al*. **2001** https://doi.org/10.1016/S0031-9422(01)00260-6
	MIC 1.56-6.25 µg/mL against 22 pathogenic bacterial strains, including 7 strains of antibiotic-resistant bacteria	**[31]** Vairappan *et al*. **2004** https://doi.org/10.1055/s-2004-832653
	Inhibition zone of 12 mm with 25 µg/disc against *S. aureus*	**[29]** Tsukamoto *et al*. **2005** https://doi.org/10.3390/md302022
	MIC < 3.9 µg/mL against marine biofilm-forming strains: *Bacillus altitudinis*, *Bacillus pumilus*, *Bacillus subtilis*, and *Bacillus cereus*	**[30]** Agúndez-Salas *et al*. **2025** https://doi.org/10.1007/s10811-024-03439-z
	MIC 25-100 µg/mL for eight *M. tuberculosis* strains, and MIC 6.2-25 µg/mL for six nontuberculous mycobacteria	**[32]** García-Davis *et al*. **2020** https://doi.org/10.3390/md18060287
Antiparasitic	IC_{50} 34.45 µM and 34.72 µM for promastigotes and amastigotes stages of *Leishmania amazonensis*, respectively	**[19]** García-Davis *et al*. **2023** https://doi.org/10.3390/md21060333
	IC_{50} 13.42 µM against *Naegleria fowleri*	**[12]** Arberas-Jiménez *et al*. **2020** https://doi.org/10.1038/s41598-020-74729-y
	IC_{50} value of 8.81 µM against cyst stage of *Naegleria fowleri*	**[33]** Arberas-Jiménez *et al*. **2022** https://doi.org/10.1128/spectrum.00515-22
Cytotoxic	EC_{50} 2.4-12.6 µg/mL against A549 (non small cell lung adenocarcinoma), SK-OV-3 (ovarian), SK-MEL-2 (skin melanoma), XF498 (CNS), and HT15 (colon) cell lines	**[36]** Ryu *et al*. **2002**
	IC_{50} = 32 µg/mL against HeLa (cervix adenocarcinoma) cell line	**[29]** Tsukamoto *et al*. **2005** https://doi.org/10.3390/md302022
	IC_{50} 67.2-165.8 µM against CHO cells (ovaries biopsy), K562 (myelogenous leukemia), MCF7 (mammary adenocarcinoma), PC3 (prostate adenocarcinoma), HeLa (cervix adenocarcinoma), A431 (epidermoid carcinoma), and A549 and NSCLC-N6 (lung cancer) cell lines	**[37]** Kladi *et al*. **2005** https://doi.org/10.1016/j.tet.2005.09.113

	IC_{50} 15.68 µg/mL for Vero and 16.07 µg/mL for MCF-7 cells	**[38]** García-Davis *et al.* **2019** https://doi.org/10.3390/md17040201
Antitumoral	Breast cancer explants treated with laurinterol at 30 µg/mL exhibited a heterogeneous response which was associated with the individual response of each human tumor sample; sensible samples showed marked necrotic cell death, while no toxic effects were observed on the resistant tumor samples	**[38]** García-Davis *et al.* **2019** https://doi.org/10.3390/md17040201
Antiviral	Inhibition of the Hepatitis B Virus (HBV) core promoter with an EC_{50} 25.5 µM	**[40]** Yamashita *et al.* **2017** https://doi.org/10.1016/j.antiviral.2017.08.001
Insecticidal	LD_{50} 2.20 µg/insect against *Reticulitermes speratus*	**[26]** Ishii *et al*. **2017**
Repellent	ED_{50} 12.65 µg/cm^2 against *Sitophilus zeamais*	**[26]** Ishii *et al*. **2017**
Larvicidal/oviposition deterrent	LC_{50} of 240 µg/mL against third-instar larvae of *Aedes aegypti*	**[41]** González-Castro *et al.* **2024** https://doi.org/10.1007/s10811-024-03194-1
Antifouling	EC_{50} 0.27 µg/mL against the settlement of the barnacle *Balanus amphitrite*<br>EC_{50} 0.65 µg/mL against *Amphibalanus amphitrite*<br>Byssal thread formation inhibition of 93% (PVA/poly(MAAc)-laurinterol system)	**[27]** Ryu and Yooh, **2003**<br>**[42]** Oguri *et al.* **2017** https://doi.org/10.3390/md15090267<br>**[44]** Osada *et al.* **2025** https://doi.org/10.1039/D5PY00263J
Acetylcholinesterase inhibition	IC_{50} 46.85 µg/mL	**[26]** Ishii *et al*. **2017**
	IC_{50} 59 µg/mL	**[41]** González-Castro *et al.* **2024** https://doi.org/10.1007/s10811-024-03194-1
Na, K-ATPase inhibition	IC_{50} 0.04 mM	**[45]** Okamoto *et al*. **2001** https://doi.org/10.1271/bbb.65.474
